# The Population Genetics of Evolutionary Rescue

**DOI:** 10.1371/journal.pgen.1004551

**Published:** 2014-08-14

**Authors:** H. Allen Orr, Robert L. Unckless

**Affiliations:** 1 Department of Biology, University of Rochester, Rochester, New York, United States of America; 2 Department of Molecular Biology and Genetics, Cornell University, Ithaca, New York, United States of America; Stanford University, United States of America

## Abstract

Evolutionary rescue occurs when a population that is threatened with extinction by an environmental change adapts to the change sufficiently rapidly to survive. Here we extend the mathematical theory of evolutionary rescue. In particular, we model evolutionary rescue to a sudden environmental change when adaptation involves evolution at a single locus. We consider adaptation using either new mutations or alleles from the standing genetic variation that begin rare. We obtain several results: *i*) the total probability of evolutionary rescue from either new mutation or standing variation; *ii*) the conditions under which rescue is more likely to involve a new mutation *versus* an allele from the standing genetic variation; *iii*) a mathematical description of the U-shaped curve of total population size through time, conditional on rescue; and *iv*) the time until the average population size begins to rebound as well as the minimal expected population size experienced by a rescued population. Our analysis requires taking into account a subtle population-genetic effect (familiar from the theory of genetic hitchhiking) that involves “oversampling” of those lucky alleles that ultimately sweep to high frequency. Our results are relevant to conservation biology, experimental microbial evolution, and medicine (*e.g*., the dynamics of antibiotic resistance).

## Author Summary

Changes to an organism's environment may have such adverse effects on fitness that the population begins to decline in size. To survive, the population must adapt before it goes extinct. Such “evolutionary rescue” is characterized by a U-shaped curve: population size declines and then recovers as a beneficial allele increases in number. Here we describe this U-shaped curve mathematically when the rescuing allele starts out rare. We obtain several results. First, we calculate when evolutionary rescue is more likely to come from new mutation than from the standing genetic variation. Second, by describing the entire U-shaped curve mathematically, we derive the time until the average rescued population begins to rebound in size as well as the smallest average population size experienced before rescue. We also find that evolutionary rescue from new mutation takes longer and involves a smaller minimum population size than rescue from the standing genetic variation.

## Introduction

The history of life is punctuated by periods of mass extinction. It has become clear that we are now living through such a period: present species extinction rates are 100–1000 fold higher than background rates [1],[2]. It is also clear that this burst of species extinction largely reflects human activity, including the combined consequences of habitat destruction, pollution, and climate change [*e.g*., [3],[1],[4],[5]]. Not surprisingly, present extinction rates— and the threat they pose to biodiversity— have received much attention over the last few decades.

Until recently, population genetics has had little to say about extinction. Extinction is, however, partly a population-genetic phenomenon. Theory as well as experiments with microbes suggest that some threatened species may be able to adapt to environmental change on a sufficiently fast time-scale to prevent their extinction. This phenomenon, so-called evolutionary rescue, has been the focus of considerable empirical and, to some extent, theoretical work [for an overview, see [6] and other papers in the special issue of the *Proceedings of the Royal Society B*].

Here we extend the population genetic theory of evolutionary rescue. We focus on a sudden environmental change that is severe enough to lower the population's mean absolute fitness below one. Consequently, the population cannot replace itself and begins to decline geometrically in numbers. Unchecked, this decline will lead to extinction. To survive, the population must adapt and it must do so quickly. As Maynard Smith [7] emphasized, adaptation in a threatened population is unlike ordinary adaptation. Instead, it is a race against extinction.

While a substantial literature considers the case in which adaptation involves a quantitative genetic (polygenic) response to selection, we consider the simple case in which adaptation involves evolution at a single locus. This case appears to be important biologically, as responses to human-induced change— *e.g*., insecticide resistance, industrial melanism, heavy metal tolerance, etc.— often involve rapid change at single genes [reviewed in [6],[8]]. We further consider an abrupt change in the environment, which then remains in this new state for the period of time that we consider [for gradual change in the environment, see reference [6]]. Finally, we focus on a particular regime in which the allele that might rescue a threatened population is initially rare, *i.e.*, either present in low copy number or appearing as a recurrent mutation. If the allele were not rare, the population would suffer little risk of extinction in the first place. Put differently, we restrict attention to that regime in which a species suffers a great risk of extinction.

**Figure 1:**
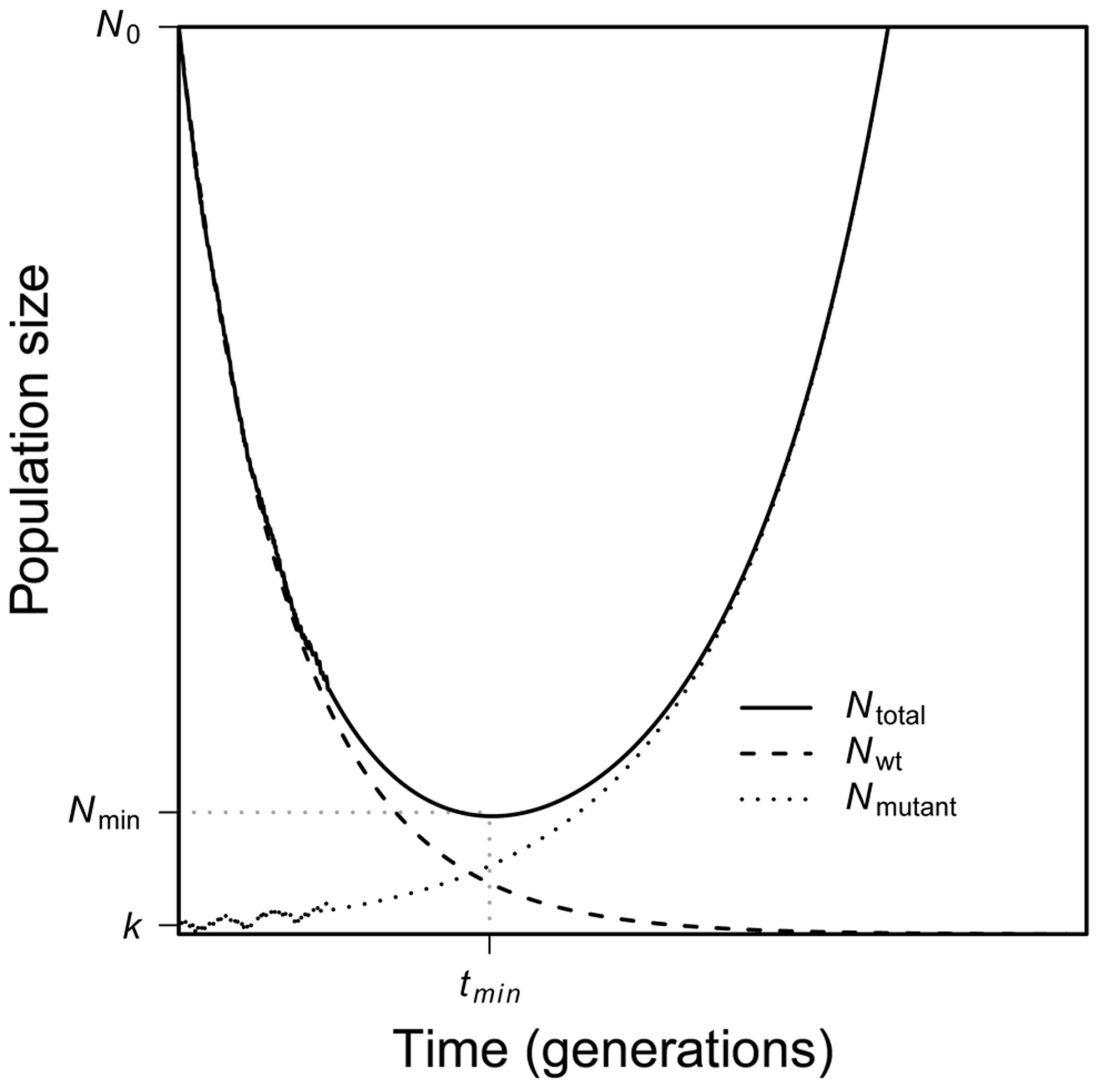
A schematic of evolutionary rescue. Following an environmental change, a population begins to decline as the wildtype suffers a fitness less than one. A rare mutant allele with fitness greater than one may increase in frequency, saving the population from extinction. Together, the two genotypes yield a characteristic U-shaped curve of total population size through time.

Evolutionary rescue is characterized by a U-shaped curve of population size [9], [10]. As Figure 1 shows, when the environment changes at time *t* = 0, mean absolute fitness drops below one, and the population begins to decline in numbers. Conditional on evolutionary rescue, mean absolute fitness will, at some point, rebound to exceed one and population size will begin to grow; this occurs at time 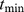. Population size will then continue to increase until attaining some large stable value. As Figure 1 also shows, the U-shaped curve for total population size is, in our scenario, the superposition of two curves: one that characterizes the geometric decline in number of individuals that carry the wildtype allele and the other that characterizes the increase in number of individuals that carry the beneficial allele.

Here our main goal is to better characterize evolutionary rescue mathematically. In particular, we describe this U-shape curve analytically. We focus on the behavior of the average size of a population through time conditional on evolutionary rescue occurring. A complete solution to this problem has, to this point, proved elusive. As we will see, part of the reason is that such a solution requires incorporating a subtle population-genetic effect (familiar from the theory of genetic hitchhiking) into this largely ecological problem.

We emphasize approximate results throughout. Given the complexity of ecological problems— all else is rarely equal in the real world— we suspect that it is more important to obtain approximate results that are intelligible and somewhat robust to departures from assumptions than exact ones that are neither. Our results are typically simple enough to allow intuitive interpretation.

## Results

### Model

We study the same model described in Orr and Unckless [11]. Briefly, we consider a haploid model in which adaptation involves a rare beneficial allele at a single locus. Mating is random and there is no population structure or migration. The environment changes suddenly, altering the fitness of alleles; these new fitness values then remain constant through the time period studied. We assume no clonal interference among beneficial alleles.

Time is discrete and measured in generations. (We will, however, make continuous time approximations when convenient.) At time *t* = 0, a population of size *N_0_* made up entirely (or almost entirely) of wildtype individuals experiences a sudden environmental change. As the wildtype allele has absolute fitness 1-*r* in the new environment, the number of wildtype individuals decreases geometrically though time. Following MacArthur and Wilson [[12], chapt. 4], Leigh [13], Lande [14], Orr and Unckless [11] and others, we assume a simple form of population regulation in which population size can grow exponentially until it hits a carrying capacity, *K*.

A beneficial allele that increases absolute fitness to 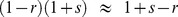 either resides at low frequency, 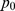, at *t* = 0 or arises recurrently by mutation after the environmental change. If the allele resides in the standing genetic variation, *k* copies are present at time *t* = 0 (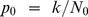). As noted in the Introduction, we assume throughout our analysis that *k* is small (though see Text S1). It seems likely that *k* might often be small in actual threatened populations as such populations often begin with fairly small sizes.

Evolutionary rescue, if it occurs, involves an increase in frequency of the beneficial allele before the population goes extinct. Any allele that can cause evolutionary rescue must enjoy an absolute fitness greater than one, requiring *s*>*r* (assuming that the product s * r is negligibly small).

One simplifying assumption that we make throughout is that the quantity *s-r* is small enough to justify Haldane's 2*s* (in our case, 2(*s-r*)) approximation to the probability that a unique mutation escapes stochastic loss. Some mutations that might save a population could be of large effect and would violate this assumption. In such cases, it is straightforward to replace the approximate quantity 2(*s-r*) in our calculations with the more exact one, 1-exp(-2(*s-r*)) throughout. The results will be more cumbersome and less intuitive but they generally do not change qualitatively.

While we are primarily interested in analytically characterizing evolutionary rescue, we check all of our approximate analytic results against computer simulations. These simulations are described in Orr and Unckless [11]. Briefly, these are exact stochastic (forward) Monte Carlo simulations that follow threatened populations of a given initial size through time.

### New mutation versus standing genetic variation

Orr and Unckless [11] calculated the probability that newly-arising mutations cause evolutionary rescue. Given a per gamete per generation rate of mutation, *u*, to a beneficial mutation of fitness effect *s* (*s*>*r*), they showed that this probability is
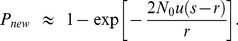
(1)


Bell [15] derived essentially the same result.

An analogous calculation lets us find the probability that alleles from the standing genetic variation cause evolutionary rescue. These alleles are present at time *t* = 0 at frequency *p_0_*. So long as these alleles are rare and each copy enjoys an independent evolutionary fate, we have
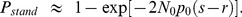
(2)


Eq. (2) is agnostic about the historical forces responsible for the presence of the allele at *t* = 0. The allele may, for instance, have been previously deleterious or previously neutral.

The total probability of evolutionary rescue from either new mutation or the standing genetic variation (we ignore rare events wherein copies of both types of alleles contribute) is 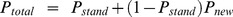 or
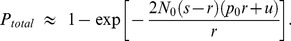
(3)


It is easy to find the conditions under which evolutionary rescue is more likely to involve the standing genetic variation *versus* a new mutation, where both types of allele enjoy selective advantage *s*. From Eq.s 1 and 2, this occurs when 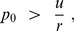
(4)a result that seems not to have been noted in the literature. New mutations are more likely than the standing variation to cause evolutionary rescue when the inequality is reversed.

**Figure 2:**
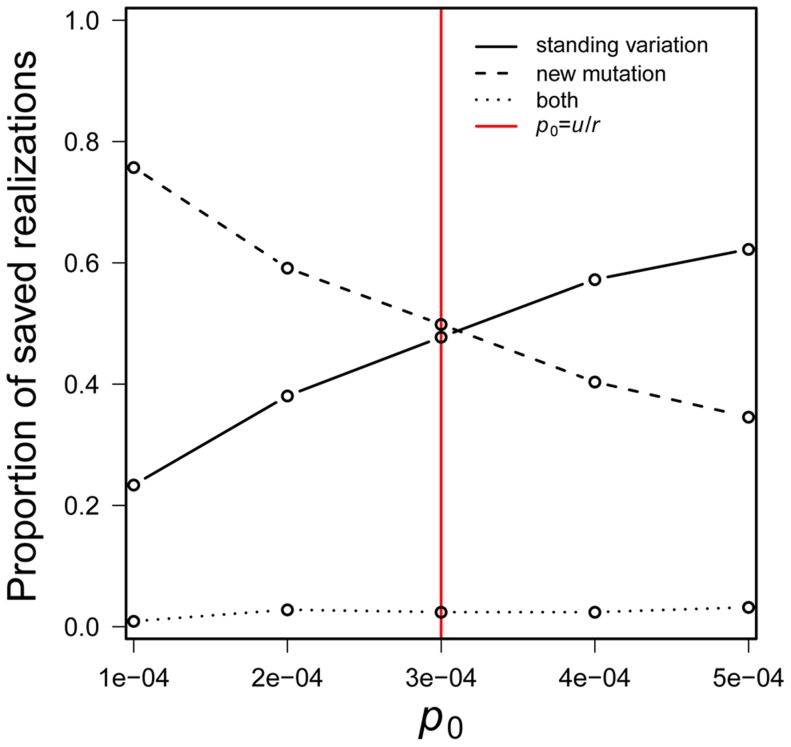
The probability that a population is saved by new mutation *versus* standing genetic variation. The standing genetic variation plays a greater role in rescue when *p*_0_ > *u/r*. Simulations assume *N_0_* = 10,000, *r* = 0.00333, *s* = 0.01 and *u* = 10^−6^ with 100, 000 realizations. Red line indicates *p*_0_ = *u/r*.

Eq. 4 is independent of both *s* and *N*_0_. Its dependence on *p_0_* and *u* is intuitive – a higher initial frequency favors a role for standing genetic variation while a higher mutation rate favors a role for new mutation. The effect of *r* is subtler. A population's rate of decline affects both standing variation and new mutation in that it decreases the rate at which the number of mutant individuals can grow (∼1-*r*+*s*). But *r* has a further effect on new mutations. Each generation, it erodes the raw material— wildtype individuals— required for production of new mutations. Thought of differently, Eq. 4 reflects the fact that the expected number of copies of the beneficial allele in the standing variation is *N*_0_ *p_0_* while the expected cumulative number produced by new mutation before a population goes extinct is *N*_0_ *u*/*r* [see reference [11]]. Given the shared factor of *N*_0_, the relative magnitudes of *p_0_ versus u*/*r* determines which scenario involves the larger number of copies. Although Eq. 4 is obviously approximate, it agrees remarkably well with computer simulations (Figure 2).

If we were to assume that standing genetic variation segregates at the deterministic mutation-selection balance (*p_0_* = *u/s_d_*; where *s_d_* is the fitness cost of the mutation before the environmental change), Eq. 4 suggests that standing genetic variation is more likely than new mutation to save the population when *u/s_d_*>*u/r*, *i.e*., when *s_d_*<*r*.

### Size of a rescued population through time: Standing variation

We now consider the U-shaped curve in Figure 1. We would like to characterize this curve mathematically, tracking the average size of a population through time conditional on evolutionary rescue. We first consider evolutionary rescue that involves a rare allele from the standing genetic variation. We derive the average population size through time (this section) as well as properties of the average rescued population at the moment that it begins to rebound (subsequent sections). We then turn to the case in which evolutionary rescue involves new mutation, which is more complex.

The total size of a population at time *t* conditional on evolutionary rescue can be written 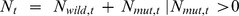, where 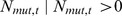 is the number of mutant individuals conditional on the allele not having been lost. Because accidental loss of a rare beneficial allele typically occurs early, this quantity can be interpreted, once *t* is appreciable, as the number of mutant individuals present conditional on evolutionary rescue ultimately occurring. Put differently, once considerable time has passed, any beneficial allele that is still present has almost certainly escaped accidental loss. Taking expectations,
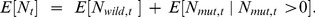
(5)


The key to our approach involves finding 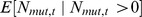. If the mutant allele were to increase in frequency deterministically, loss would never occur and we would have 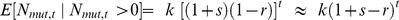. Consequently, it *might* seem that the expected population size would be
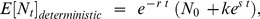
(6)where we use a continuous time approximation and that, in continuous time, 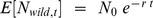. We also assume that *k* is small enough that the initial number of wildtype individuals is ∼*N_0_*. Eq. 6 essentially reflects the approach of Gomulkiewicz and Holt [9], although their single-locus model was diploid and featured Malthusian fitness parameters.

While Eq. 6 is adequate when both alleles are common, it can diverge dramatically from the correct solution when the rescuing allele is initially present in low copy number (see below). The source of the discrepancy is simple. Loss of rare beneficial alleles is common and the above approach ignores this loss. More subtly, loss of rare beneficial alleles affects not only the probability of evolutionary rescue but expected population size when the beneficial allele is *not* lost. The point was seen by Maynard Smith [16] and emphasized by Maynard Smith and Haigh [17] in their classic analysis of genetic hitchhiking.

The key point is that successful alleles— those that sweep to fixation— are not a random sample of initially-rare beneficial alleles. Instead, successful alleles are disproportionately those that rise by genetic drift to higher than expected copy number in the first few generations of their evolutionary histories [18]. Such alleles have a greater chance of being successfully “grabbed” by natural selection. Maynard Smith showed that this oversampling effect could be taken into account in otherwise-deterministic selection equations by a simple, albeit approximate, approach. It is, he argued, as though the alleles that successfully fix began with higher copy number than they actually did, a finding that often plays a part in hitchhiking theory. [This point is also well known in the branching process literature, at least in certain limiting cases, *e.g*., [19]]

This insight can be imported into our problem to find 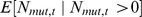. Here we follow Maynard Smith's [16] informal argument. (He considers a unique new mutation but, as we will see, his argument is trivially generalized to a rare allele from the standing variation.) Maynard Smith noted that, with *t* appreciable enough that a mutant allele has either been lost or has reached large enough numbers that it will ultimately fix, we have 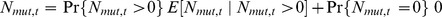, where the left hand side is the number of mutant individuals expected deterministically and 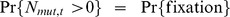. Thus
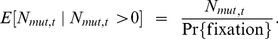
(7)


In the case of a new mutation in a stable population, 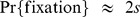 and, conditional on non-loss, the expected number of individuals that carry the beneficial allele at time *t* is larger by a factor of 1/(2*s*) than expected naively [17].

Our problem differs from Maynard Smith's in two small ways. First, our allele begins from the standing variation. Second, our population is shrinking. Both effects can be taken into account to calculate the appropriate 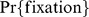 in Eq. 7. Given *k* copies at time *t* = 0,
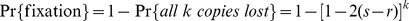
(8)where 2(*s*-*r*) is the approximate probability of fixation of a unique copy of the beneficial allele with small selective advantage in a population that declines geometrically [20]. For an allele that starts at low copy number, *i.e*., *k* is small, Eq. 8 is, to a good approximation, 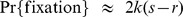. Thus Eq. 6 becomes 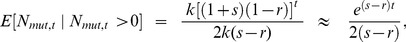
(9)where the last step is a continuous time approximation appropriate with small *s*-*r*.

Perhaps surprisingly, Eq. 9 is independent of *k* (for small *k*). In words, the expected number of mutant individuals at time *t* conditional on ultimate fixation equals the deterministic expectation for a *single new mutation* normalized by its probability of fixation. This result has a simple interpretation. When starting with small *k* and conditioning on fixation, descendants of only one copy typically sweep to fixation. (This reflects the fact that the probability of fixation of each copy is generally small with weak selection, especially in a declining population.) The expected number of copies present at time *t* conditional on ultimate fixation is therefore the same as that for a single mutation normalized by its probability of fixation.

Substituting in Eq. 5, the expected total size of a population at time *t* conditional on evolutionary rescue is
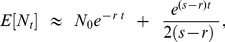
(10)where we again use a continuous time approximation with 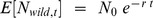 in continuous time. (We also again assume that the initial number of mutant individuals is negligible compared to *N_0_*.)

**Figure 3:**
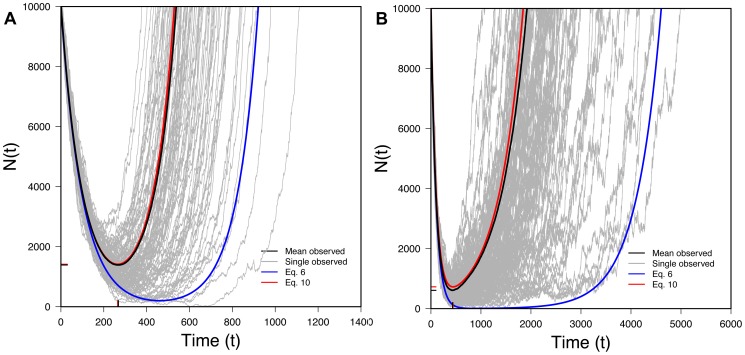
The U-shaped trajectory for populations rescued from standing variation. 100 randomly selected successful realizations in gray, mean of all successful realizations in black, Eq. 6 (without the oversampling correction) in blue, Eq. 10 (with the oversampling correction) in red. Ticks on X-axis represent observed (black) and predicted (red) *t_min_* (Eq. 13) while ticks on the Y-axis represents observed (black) and predicted (red) *N_min_* (Eq. 14). A) *N_0_* = 10,000, *r* = 0.01, *s* = 0.02, *k* = 1; B) *N_0_* = 100,000, *r* = 0.01, *s* = 0.02, *k* = 1; 100,000 realizations.

Eq. 10 is one of our key results. It lets us trace the expected size of a rescued population through time. Figure 3 shows that Eq. 10 performs very well when compared to exact computer simulations. Figure 3 also shows that Eq. 6, which ignores Maynard Smith's oversampling effect, performs poorly when the desired beneficial allele is initially present as a small number of copies.

We can also find the variance in 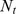 conditional on evolutionary rescue, at least roughly. Given that the two types propagate independently, 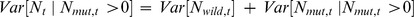. If we assume the wildtype population is large enough that it behaves approximately deterministically (an assumption that will break down at some point), most of the variance in 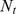 will reflect variance in *N_mut_*. Crudely, then, 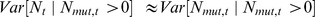. Theory extending Maynard Smith's insight shows that, conditional on fixation and with *t* large, the distribution of copy number for a beneficial allele that escapes loss is approximately exponential with the mean given in Eq. 9 [18], [19]. Thus 
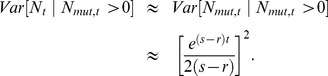
(11)


**Figure 4:**
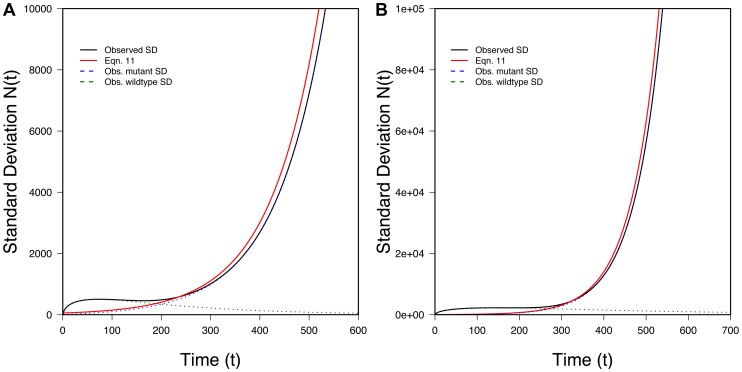
The variance in population size during evolutionary rescue. Observed variance of total population size (black), predicted from Eq. 11 (red), variance in the number of mutants (dotted blue), variance in number of wildtype (dotted green). A) *N_0_* = 10,000, *r* = 0.01, *s* = 0.02, *k* = 10; B) *N_0_* = 100,000, *r* = 0.005, *s* = 0.02, *k* = 3; 100;000 realizations.

Figure 4 shows that this approximation performs well once *t* is appreciable. The fit is less good early on.

Text S1 generalizes the results of this section when the number, *k*, of copies of the beneficial allele present at *t* = 0 is not small.

### Population rebound: Standing variation

We now characterize the average rescued population at the moment it begins to rebound in size, *i.e.*, the moment it hits its minimal size in Figure 1. When does this rebound occur? And what is the smallest size, 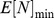, experienced by the average rescued population?

To find the time the mean population begins to rebound, note from Eq. 10 that 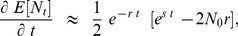
(12)which equals zero when
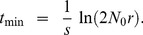
(13)


**Figure 5:**
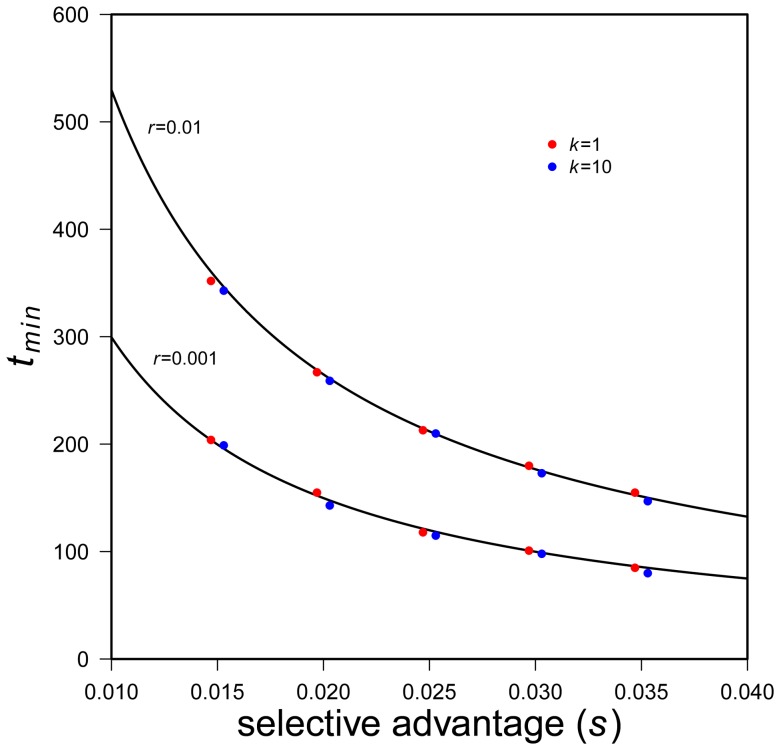
Time to minimum expected population size. Black lines are predicted *t_min_* (Eq. 13), simulation results with *k* = 1 are red dots, simulation results with *k* = 10 are blue dots. Two values of *r* are used (see plot), *N_0_* = 10,000. 5000 successful realizations for each set of parameters.

As 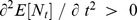, Eq. 13 represents a minimum, which can also be seen in Figure 3. Figure 5 also shows that Eq. 13 is very accurate.

Eq. 13 shows, not surprisingly, that the greater selective advantages of a mutant allele the shorter the time to rebound. (It may seem surprising that *s* alone, not *s*-*r*, appears. The reason is that the system of allele frequency changes depends only on the fitness *difference* between genotypes.) The effect of *r* is harder to intuit. Eq. 13 shows that, all else equal, faster population decline increases the time until expected *N* rebounds. To understand this, note that Eq. 13 represents a time that is conditional on rescue. With larger *r* the probability of such rescue decreases but— if rescue does occur— larger *r* slows the time to rebound. The reason is that the absolute number of mutant individuals grows as ∼1+*s*-*r*, which is smaller for larger *r*. It is also worth noting that the time until rebound is independent of copy-number, *k*, of the beneficial allele, so long as *k* is small. This can also be seen in Figure 5. (See Text S1 for results with somewhat larger *k*.) Eq. 13 also makes clear that the time until rebound is more sensitive to *s* than to *N_0_* and *r*, which enter only logarithmically.

We can also find the minimum expected population size experienced during evolutionary rescue. Substituting *t_min_* into Eq. 10, we get 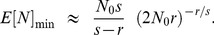
(14)


Figure 3 shows that Eq. 14 performs well compared to simulations. The size of the average population at its minimum will obviously affect a population's genetic diversity post-recovery as effective population size is sensitive to the smallest population size experienced through time.

Our approach in this section has involved characterizing the expected size of a population, 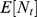, conditional on rescue. Eq. 13, for instance, gives the time when 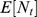 hits its minimum; this is not necessarily identical to the average of the times when individual realizations hit their minimum. Similarly, Eq. 14 gives the minimum size of 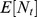 conditional on rescue; this is not necessarily identical to the average of the minima experienced in individual realizations. (The minimum of the average need not equal the average of the minima.) Nonetheless, Figure 3 shows that our approach roughly captures the behavior of what might be loosely thought of as the “typical” rescued population.

We can also specify relations between the numbers of mutant and wildtype individuals present when the average population rebounds. At *t_min_* we find that 
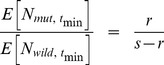
(15)to the order of our approximations. This result is independent of *N_0_* and *p_0_*, so long as *k* is small.

Simulations show that Eq. 15 is reasonably accurate. For example, with *s* = 0.02 and *r* = 0.001, 0.005 or 0.01, Eq. 15 yields 0.053, 0.333 and 1.00, respectively. The observed values were 0.054, 0.316 and 0.923 given an initial population size of *N_0_* = 10,000 and *k* = 1 (100,000 successful realizations). If we increase the initial population size to *N_0_* = 100,000 (with *k* = 1), the observed values were 0.054, 0.347 and 1.05 (100,000 successful realizations). If, on the other hand, we increase the number of copies of the mutant alleles segregating in the standing variation, *i.e*., *k* = 10 and *N_0_* = 10,000, the observed values are 0.051, 0.331, 0.951 (100,000 successful realizations).

### Population return to carrying capacity: Standing variation

We can also estimate the time needed for the average population to return to its pre-crisis carrying capacity, *N_0_*. This occurs when Eq. 10 equals *N_0_*, which is roughly
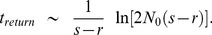
(16)


This approximation is crude in several ways. First, we assume that the number of wildtype individuals is negligible by the time the population arrives at 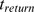.

Second, we assume that the carrying capacity in the new environment is the same as in the old environment. This need not be true and there is some experimental evidence that it is not, at least in the laboratory [21]. Third, we assume, following MacArthur and Wilson [12] and Lande [14] and others, that the population maintains exponential growth until hitting carrying capacity. Eq. 16 would almost certainly be inappropriate under other forms of population regulation.

### Size of a rescued population through time: New mutation

We now turn to characterizing the U-shaped curve when evolutionary rescue involves a new mutation. This scenario is more complex than above as we must consider two dynamics: the time until a successful new mutation arises and the time then required for the allele to reach high frequency.

In particular, we modify the approach taken above to reflect a delay in the time required for a new mutation to appear that escapes stochastic loss. During this delay the number of wild-type individuals continues to decline. Consequently, the mean population size conditional on rescue will behave as:
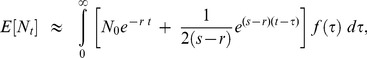
(17)where 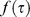 is the probability density of waiting times for the origin of a successful new mutation. The quantity 2(*s*-*r*) in the denominator again takes Maynard Smith's oversampling effect into account.

Orr and Unckless [11] showed that the distribution of waiting times until the appearance of a new mutation that escapes loss in a geometrically declining population is itself approximately geometric (∼ exponential):
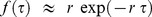
(18)


In words, because the population declines geometrically, the supply of new mutations declines about geometrically and, consequently, rescue is more likely early than late. Although improved solutions to the distribution of origination times of successful mutations are possible (see Text S2, Figure S1), we rely here on Eq. 18, which is simple and often adequate.

From Eq.s 17 and 18, we find that the expected population size is
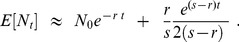
(19)


**Figure 6:**
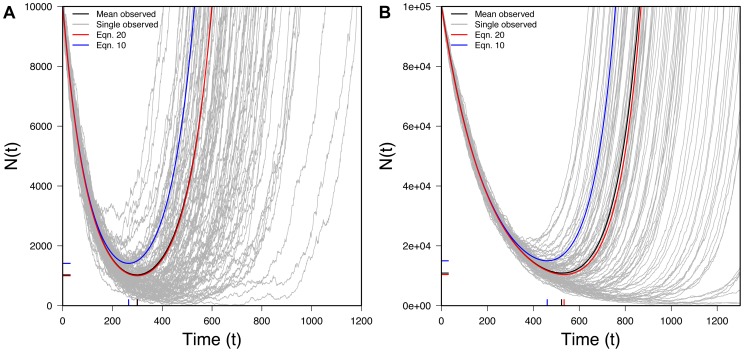
The U-shaped curve for populations rescued by new mutation. 100 randomly selected successful realizations in gray, mean of all successful realizations in black, Eq. 19 (expectation from new mutation) in red, Eq. 10 (expectation from standing variation) in blue. Ticks on X-axis represent observed (black) and predicted (red) *t_min_* (Eq. 21) while ticks on the Y-axis represents observed (black) and predicted (red) *N_min_* (Eq. 22). A) *N_0_* = 10,000, *r* = 0.01, *s* = 0.02, *u* = 10^−5^ B) *N_0_* = 100,000, *r* = 0.005, *s* = 0.015, *u* = 10^−6^; 10,000 realizations.

Figure 6 shows that Eq. 19 performs very well compared to simulations. Figure 6 also shows 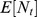 when alleles derive from the standing variation (Eq. 10), allowing comparison between the two scenarios. With new mutation, the U-shaped curved is stretched to the right: recovery takes longer than with standing variation, reflecting the waiting time required for the appearance of a successful new mutation.

Interestingly, the expected size of a rescued population under new mutation (Eq. 19) is identical to that under standing variation (Eq. 10) except for the factor of *r*/*s* in the second term of Eq. 19. Because, with rescue, *r*/*s*<1, the mean size of a rescued population is, at any moment, smaller with new mutation than with standing variation, reflecting the waiting time for the appearance of a successful new mutation.

By analogy to Eq. 11, and continuing to ignore the variance in numbers of wildtype individuals, the variance in population size at time *t* is
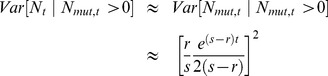
(20)


Simulations confirm that Eq. 20 performs well when *t* is not very small (not shown).

We can also solve for the time at which the mean population begins to rebound, conditional on rescue by new mutation. It is:
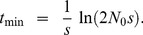
(21)


As Figure 6 shows, this result is quite accurate. Remarkably, *t_min_* is independent of *r*, to the order of our approximations. This is because *r* plays two opposing roles in the time to E[*N*]_min_. First, as *r* increases (with *s* constant), the rate of increase of the mutant allele (with fitness 1+*s-r*) decreases. This increases the time to E[*N*]_min_. Second, as *r* increases, the population declines faster, so that— conditional on rescue occurring— the new mutation must have arisen fairly soon after the environmental change.

As remarkably, *t_min_* with new mutation is identical to that with standing variation except that the quantity *s* replaces *r* in the argument of the logarithm (see Eq. 13). Because *s*>*r*, *t_min_* for new mutation is larger than that for standing variation, again reflecting the additional waiting time for the appearance of a successful new mutation.

Finally, the mean population size at *t_min_* with new mutation is
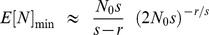
(22)


Not surprisingly, this is smaller than the mean size found for standing variation (Eq. 14). Because rebound occurs later with new mutation, populations decline to a smaller average size before rebounding.

We can also estimate the time needed for the average population to return to its pre-crisis carrying capacity. This occurs when Eq. 10 equals *N_0_*, which yields roughly
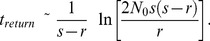
(23)


This equation is identical to that for the standing variation case (Eq. 16) except, again, for the term of *s*/*r* in the argument of the logarithm; consequently, 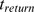 is longer for new mutation than it is for standing variation. The same (serious) caveats apply to Eq. 23 as to Eq. 16.

## Discussion

We have extended the population-genetic theory of evolutionary rescue. In particular, we have considered a scenario in which the environment changes suddenly and the population attempts to adapt to this change via evolution at a single locus. (Though far from universal, considerable data indicate a role for single genes in response to, *e.g*., human disturbance; see below.) We further focus on a particular regime in which the desired beneficial allele is initially rare, *i.e*., it is either present in few copies at time zero or it appears as a new mutation after time zero. This regime is of special interest as adaptation is far from deterministic and the population is therefore seriously threatened by the environmental change and suffers a considerable probability of extinction. If the beneficial allele were much more common, adaptation would be essentially deterministic and the population would suffer little probability of extinction. Gomulkiewicz and Holt [9] considered this case in which natural selection at a single locus deterministically rescues a threatened population. (There is obviously a gray area between the rare and common allele regimes; see Text S1 for some analysis of this gray area.)

One of our most interesting findings involves comparing evolutionary rescue from a rare allele that resides in the standing genetic variation *versus* a new mutation. Eq. 4 shows that rescue from the standing variation is more likely than that by new mutation when the initial frequency, *p*_0_, of the beneficial allele exceeds *u*/*r*. Conversely, new mutation is more likely to be involved when *p*_0_<*u*/*r*. These results are independent of *s*— given that both types of alleles share the same selective advantage— and reflect the relative expected number of copies of the desired allele that arise by new mutation before extinction *versus* that reside in the standing variation.

We have also derived an approximate equation for the U-shaped curve that characterizes evolutionary rescue (Eqs. 10 and 19). This curve describes the trajectory of expected population size through time conditional on rescue. When beneficial alleles are initially rare (small *k*), derivation of this quantity requires taking into account a subtle population-genetic effect familiar from the theory of genetic hitchhiking: rare beneficial alleles that sweep to high frequency behave, in deterministic selection equations, as though they began at a higher frequency than they actually did. The reason, first seen by Maynard Smith [16], is that natural selection is more likely to “choose” alleles that accidentally drift to somewhat higher than expected frequencies early in their histories [see also [18]]. Incorporating this oversampling effect into our calculations, we derive several key quantities that characterize the U-shaped curve of evolutionary rescue, including the time until the expected population size begins to rebound (Eq.s 13 and 21) and the smallest expected population size experienced (Eq.s 14 and 22). These quantities assume surprisingly simple forms and perform well when compared to exact computer simulations given small *k*.

The U-shaped curves of evolutionary rescue differ depending on whether rescue involves new mutation (Eq. 19) or rare alleles from the standing variation (Eq. 10). In particular, the waiting time until the mean population size begins to rebound is longer with new mutation than with standing variation. Similarly, the minimum expected population size experienced during rescue is smaller with new mutation than with standing variation. Both findings reflect the fact that the U-shaped curve for rescue is delayed, *i.e*., stretched to the right, for new mutation relative to standing variation. This, in turn, reflects that evolutionary rescue by new mutation involves a waiting time that does not appear with the standing variation— that required for the appearance of a successful mutation, *i.e*., one that escapes accidental loss. We also derive the variance in population size through time, albeit roughly.

Though beyond the scope of the current paper, our results have some implications for the genetic diversity of populations that experienced evolutionary rescue from a sudden environmental shock. For example, as the minimum average population size is always smaller under evolutionary rescue from new mutation than from the standing genetic variation, populations that adapted via a new mutation will likely often experience more loss in diversity than populations that adapted from the standing variation. (It must be emphasized, however, that our results involve the expected size of rescued populations and there is often much variation about these expected values.)

Our analysis does feature several important limitations. First, we focus on adaptation at a single locus. While the frequency of single-locus adaptation (or, more plausibly, adaptation that involves a major effect at some single locus) remains somewhat uncertain, matters are clearer when considering sudden and dramatic environmental changes of the sort modeled here. Many such changes, or at least those that have been analyzed genetically, involve responses to human disturbance, *e.g*., antibiotic resistance, insecticide resistance, industrial melanism, etc. These environmental challenges are often met via evolution at a single locus [reviewed in [6],[8]]. We suspect, then, that our results, while limited, are relevant to fairly sudden evolutionary rescue from human-induced change in the environment. Second, we have focused on rescuing alleles that are initially rare, *i.e*., either residing at low copy-number in the standing genetic variation or arising recurrently by mutation. Although this need not be the case, evolutionary rescue of seriously threatened species will likely often involve rarer rather than more common alleles as species would not be much threatened to begin with if the rescuing allele were common at the moment of environmental change. In any case, the population-genetic theory of rescue in the case of common alleles is straightforward as it is essentially deterministic [9].

Third, our analytic findings are approximate. This partly reflects the difficulty of the problem considered and partly our attempt to uncover simple patterns that might characterize rescue. Given the complexities of ecological change in the real world, we suspect that it may be most useful, at least at this stage in our understanding, to obtain theoretical results that are approximate but intelligible than ones that are exact but difficult to intuit. In any case, our analytic results generally perform well when compared to computer simulations. We thus believe therefore that our findings represent reasonable rules of thumb that characterize evolutionary rescue when adaptation involves a single gene.

While we have couched our results in terms of conservation biology, it is worth noting that they are also relevant to a medical context. Consider, for example, a pathogen population (*e.g*., a bacterial infection in a patient) that suddenly encounters a changed environment (*e.g*., antibiotics) in its host. In such medical contexts, one obviously hopes to *avoid* evolutionary rescue of the pathogen, ensuring that medical intervention drives the pathogen to extinction. Because our approach employs branching process theory, any threatened population has only two ultimate fates: adaptation or extinction. As the probabilities of these events are complementary, our analysis is obviously relevant both to conservation biology and to medical intervention against pathogens. In particular, the conditions under which we are likely to avoid adaptation of a pathogen to medical intervention are the same as those under which the probability of evolutionary rescue in our calculations is minimized.

Finally, empirical testing of our findings should be straightforward in the context of microbial experimental evolution research. Indeed several studies have reported experiments that resemble those needed to test our theory [*e.g*., [21],[22],[23]]. While more replication would be needed to determine, for example, the consequences of the oversampling effect and new quantities, *e.g*., *t_min_* and *N_min_*, would need to be measured, we see no principled problem with such direct experimental tests.

## Supporting Information

Figure S1 The distribution of waiting times until a new beneficial mutation arises that escapes stochastic loss and rescues the population. Gray bars represent the observed distribution from 10,000 successful realizations, blue line is the predicted approximate exponential distribution (Eq. 18), red line is the improved distribution (Eq. S2.3). A) *N_0_* = 10,000, *r* = 0.01, *s* = 0.02, *u* = 10^−5^ B) *N_0_* = 10,000, *r* = 0.003, *s* = 0.02, *u* = 10^−5^.
(PDF)

Text S1 Derivation of analytical results for the U-shaped curve of evolutionary rescue when the number of starting mutants (*k*) is not small.
(DOCX)

Text S2 Derivation of the distribution of waiting times until a new mutation arises that eventually saves the population.
(DOCX)

## References

[1] Pimm SL, Russell GJ, Gittleman JL, Brooks TM (1995) The future of biodiversity. Science 269: 347–350. 17841251 10.1126/science.269.5222.347

[2] Leakey RE, Lewin R (1994) The sixth extinction: patterns of life and the future of humankind. New York: Doubleday.

[3] Tilman D, May RM, Lehman CL, Nowack MA (1994) Habitat destruction and the extinction debt. Nature 371: 65–66.

[4] Pimm SL, Raven P (2000) Biodiversity: extinction by the numbers. Nature 403: 843–845. 10706267 10.1038/35002708

[5] Thomas CD, Cameron A, Green RE, et al (2004) Extinction risk from climate change. Nature 427: 145–148. 14712274 10.1038/nature02121

[6] Gonzalez A, Ronce O, Ferriere R, Hochberg ME (2013) Evolutionary rescue: an emerging focus at the intersection between ecology and evolution. Philos Trans R Soc Lond B Biol Sci 368: 20120404 doi:10.1098/rstb.2012.0404 23209175 10.1098/rstb.2012.0404 PMC3538460

[7] Maynard Smith J (1989) The causes of extinction. Philosophical Transactions of the Royal Society B: Biological Sciences 325: 241–252. 10.1098/rstb.1989.0086 2574882

[8] Ffrench-Constant RH, Daborn PJ, Le Goff G (2004) The genetics and genomics of insecticide resistance. Trends in Genetics 20(3): 163–70. 15036810 10.1016/j.tig.2004.01.003

[9] Gomulkiewicz R, Holt RD (1995) When does evolution by natural selection prevent extinction? Evolution 49: 201–207. 10.1111/j.1558-5646.1995.tb05971.x 28593677

[10] Holt RD, Gomulkiewicz R (2004) Conservation implication of niche conservatism and evolution in heterogeneous environments. In Ferriere R, Dieckmann U, Couvert D, editors. Evolutionary Conservation Biology. Cambridge: Cambridge University Press. pp.244–264.

[11] Orr HA, Unckless RL (2008) Population extinction and the genetics of adaptation. American Naturalist 172: 160–169. 10.1086/589460 18662122

[12] MacArthur RH, Wilson EO (1967) The theory of island biogeography. Princeton: Princeton University Press.

[13] Leigh EG Jr (1981) The average lifetime of a population in a varying environment. Journal of Theoretical Biology 90: 213–239. 7311579 10.1016/0022-5193(81)90044-8

[14] Lande R (1993) Risk of population extinction from demographic and environmental stochasticity and random catastrophes. American Naturalist 142: 911–927. 10.1086/285580 29519140

[15] Bell G (2008) Selection, the Mechanism of Evolution. 2^nd^ edition. Oxford: Oxford University Press.

[16] Maynard Smith J (1971) What use is sex? J Theor Biol 30: 319–335. 5548029 10.1016/0022-5193(71)90058-0

[17] Maynard Smith J, Haigh J (1974) The hitch-hiking effect of a favourable gene. Genetical Research 23: 23–35. 4407212

[18] Barton NH (1995) Linkage and the limits to natural selection. Genetics 140: 821–841. 7498757 10.1093/genetics/140.2.821 PMC1206655

[19] Fahady KS, Quine MP, Vere-Jones D (1971) Heavy traffic approximations for the Galton-Watson process. Adv Appl Prob 3: 282–300.

[20] Otto SP, Whitlock MC (1997) The probability of fixation in populations of changing size. Genetics 146: 723–733. 9178020 10.1093/genetics/146.2.723 PMC1208011

[21] Bell G, Gonzalez A (2009) Evolutionary rescue can prevent extinction following environmental change. Ecology Letters 12: 942–948. 19659574 10.1111/j.1461-0248.2009.01350.x

[22] Ramsayer J, Kaltz O, Hochberg ME (2013) Evolutionary rescue in populations of *Pseudomonas fluorescens* across an antibiotic gradient. Evolutionary Applications 6 (4): 608–616. 23789028 10.1111/eva.12046 PMC3684742

[23] Martin, G Aguilée R, Ramsayer J, Kaltz O, Ronce O (2013) The probability of evolutionary rescue: towards a quantitative comparison between theory and evolution experiments. Phil Trans R Soc B 2013 368: 1471–2970. 10.1098/rstb.2012.0088 PMC3538454 23209169

