# Supplementary figures and images for: The Population Genetics of Evolutionary Rescue
Source: PLoS Genet. 2014 Aug 14;10(8):e1004551. doi: 10.1371/journal.pgen.1004551 (PMC4133041; doi:10.1371/journal.pgen.1004551)

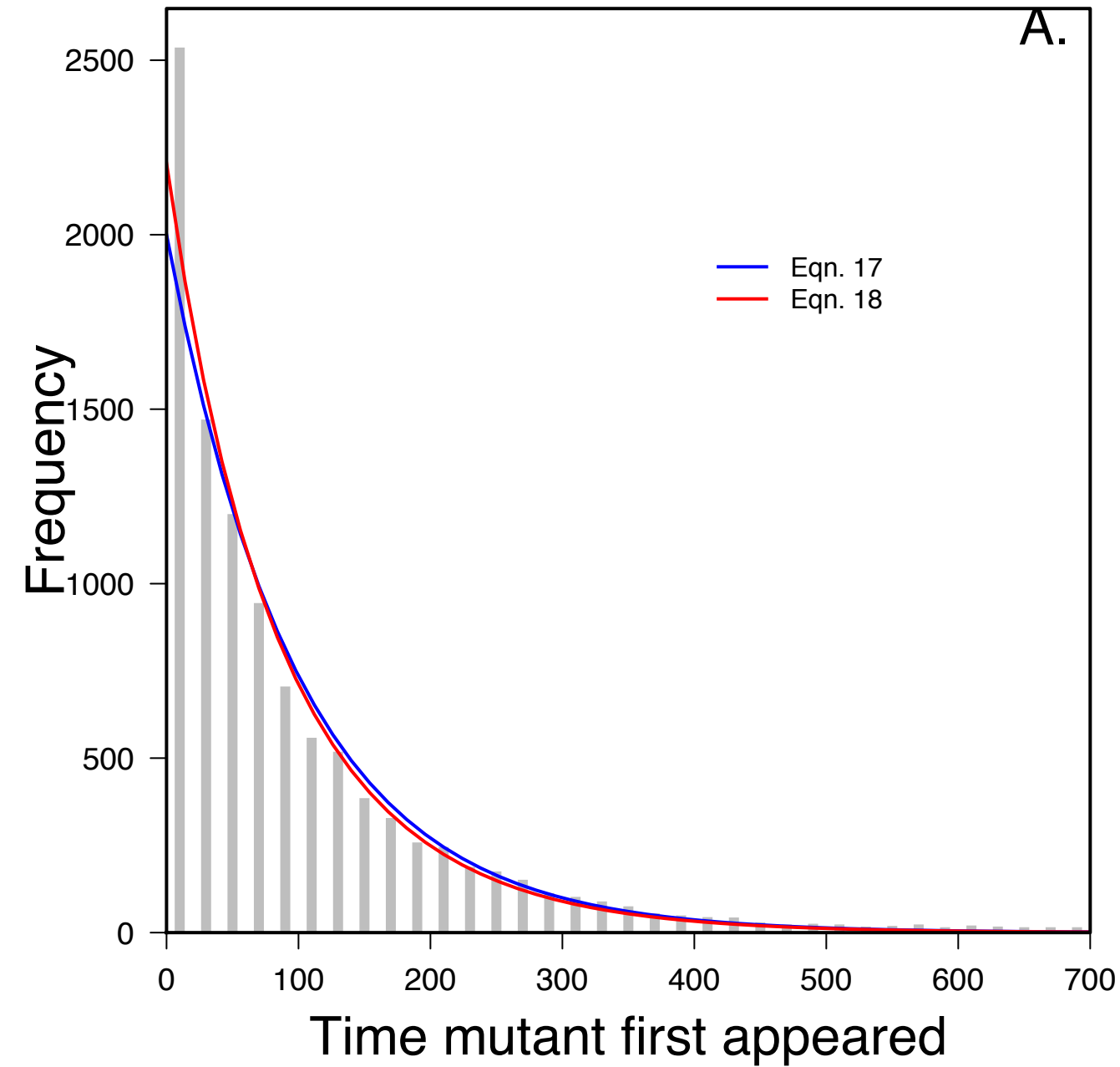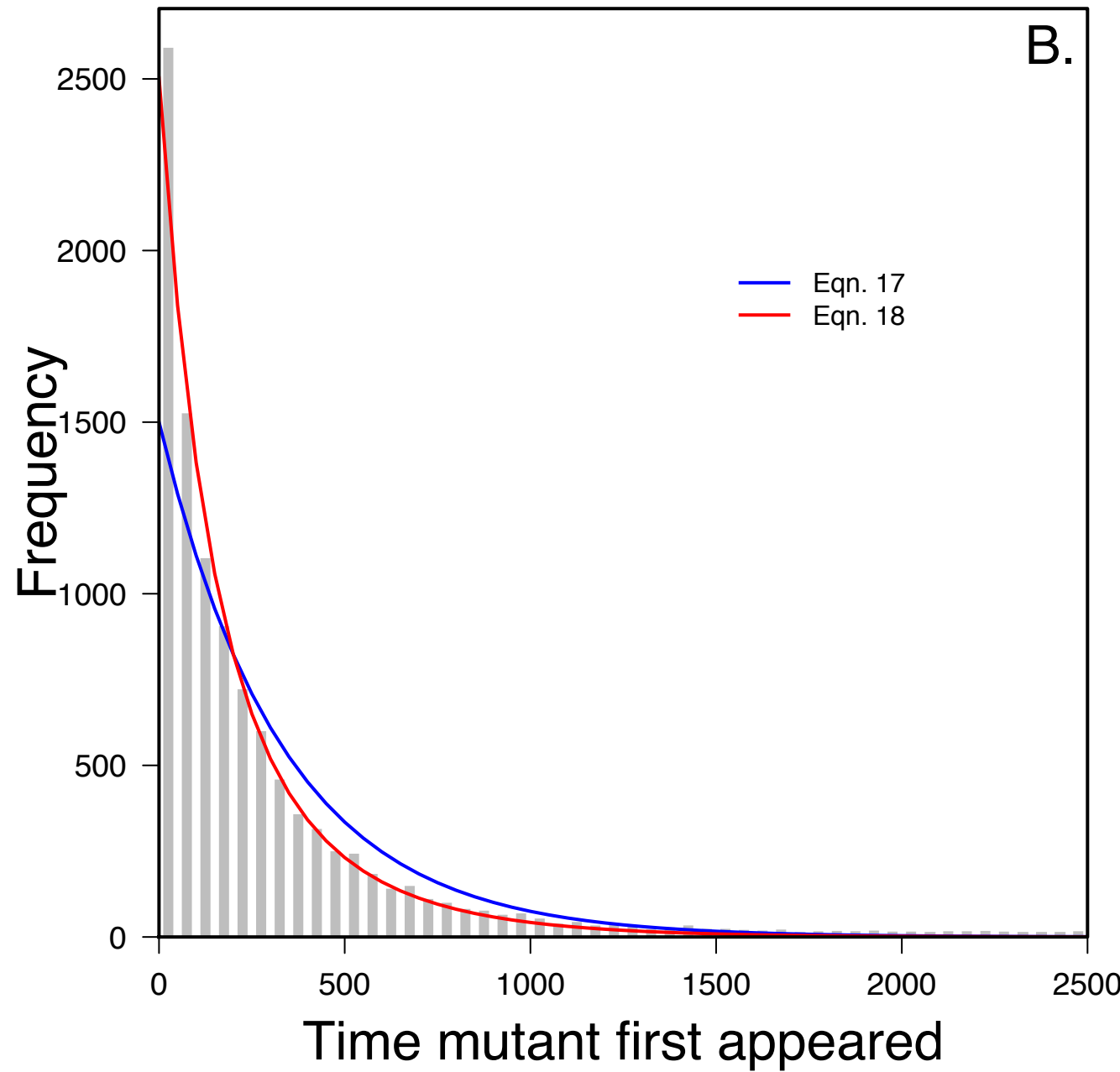

Supplement: Figure S1 — The distribution of waiting times until a new beneficial mutation arises that escapes stochastic loss and rescues the population. Gray bars represent the observed distribution from 10,000 successful realizations, blue line is the predicted approximate exponential distribution (Eq. 18), red line is the improved distribution (Eq. S2.3). A) N0 = 10,000, r = 0.01, s = 0.02, u = 10−5 B) N0 = 10,000, r = 0.003, s = 0.02, u = 10−5. (PDF) [file pgen.1004551.s001.pdf]
