# Supplementary material for: The Population Genetics of Evolutionary Rescue
Source: PLoS Genet. 2014 Aug 14;10(8):e1004551. doi: 10.1371/journal.pgen.1004551 (PMC4133041; doi:10.1371/journal.pgen.1004551)
Supplement: Text S1 — Derivation of analytical results for the U-shaped curve of evolutionary rescue when the number of starting mutants (k) is not small. (DOCX) [file pgen.1004551.s002.docx]

**Text S1**

Here we generalize our results on the trajectories of rescued population allowing that the initial number of copies, *k*, of the now-beneficial allele may not be very small. (We do assume, however, that *k* remains small relative to *N_0_*. *I.e*., the mutant allele is much rarer than the wildtype at *t* = 0. Otherwise, the population would likely not be threatened by the environmental change in the first place.) Our calculations remain simple although the results are less elegant.

As before,

$E\left[N_{t}\right] = E\left[N_{wild,t} \right] + E\left[N_{mut,t} | N_{mut,t} >0\right]$ (S1.1)

and

. (S1.2)

From Eq. 8, we see that

$\begin{matrix} \Pr\left\{\text{fixation}\right\} = 1-\Pr\left\{all k copies lost\right\} \\ = 1-\left[1-2(s-r)\right]^{k} \\ \\ \approx1-\exp\left[-2k\left(s-r\right)\right], \end{matrix}$ (S1.3)

where we no longer assume that *k* is very small but still assume small *s*-*r*.

Substituting,

$E\left[N_{t}\right] \approx N_{0} e^{-r t} + \frac{k e^{\left(s-r\right)t}}{1-\exp\left[-2k\left(s-r\right)\right]}$, (S1.4)

where we assume that *k* is sufficiently modest that the initial number of wildtype individuals is ~ *N_0_*.

As the frequency of the beneficial allele at *t* = 0 is $p_{0} = k/N_{0}$, Eq. S1.4 can be re-written

$E\left[N_{t}\right] \approx N_{0} e^{-r t} \left\{1 + \frac{p_{0} e^{s t}}{1-\exp\left[-2N_{0}p_{0}\left(s-r\right)\right]}\right\}$, (S1.5)

where, for simplicity, we let $1-p_{0} \approx1$.

Eqs. S1.2 and S1.3 also yield a rough approximation of the variance in $N_{t}$ conditional on rescue:

$Var\left[N_{t}\right] \approx\left\{\frac{k e^{\left(s-r\right)t}}{1-\exp\left[-2k\left(s-r\right)\right]}\right\}^{2}$ (S1.6)

The above results can be used to calculate more exactly statistics like the time until the average rescued population begins to rebound in size. In particular, differentiation of Eq. S1.4 shows that

$t_{\min} \approx\frac{1}{s} Log\left[\frac{N_{0}r \left(1-e^{-2k\left(s-r\right)}\right)}{k\left(s-r\right)}\right]$. (S1.7)

As *k* gets very small, this approaches $t_{\min} \approx\frac{1}{s} \ln\left(2N_{0}r\right)$ , as expected from the text (Eq. 13).

Similarly, the minimum expected population size experienced during evolutionary rescue is

$E\left[N\right]_{\min} \approx\frac{N_{0}s}{s-r} \left[\frac{N_{0}r \left(1-e^{-2k\left(s-r\right)}\right)}{k\left(s-r\right)}\right]^{-r/s}$, (S1.8)

which approaches Eq. 14 of the text as *k* gets very small.
