# Supplementary material for: The Population Genetics of Evolutionary Rescue
Source: PLoS Genet. 2014 Aug 14;10(8):e1004551. doi: 10.1371/journal.pgen.1004551 (PMC4133041; doi:10.1371/journal.pgen.1004551)
Supplement: Text S2 — Derivation of the distribution of waiting times until a new mutation arises that eventually saves the population. (DOCX) [file pgen.1004551.s003.docx]

**Text S2**

Orr and Unckless [1] showed that the waiting time until the origination of a new mutation that eventually sweeps and fixes in a population is roughly exponential with rate *r*. While this approximation is often adequate, it can be improved upon.

To see this, note that the cumulative probability that a new mutation that has arisen by generation *t* saves the population is 1 minus the probability that all mutations that have appeared by *t* are lost. In symbols,

$P_{T\leq t} = 1-\left[1-2\left(s-r\right)\right]^{A}$, (S2.1)

where $2\left(s-r\right)$ is the probability of fixation of a unique mutation in a geometrically declining population and *A* is the cumulative number of mutations that have appeared by *t*. From Orr and Unckless [1] we know that, in a geometrically (~exponentially) declining population, $A \approx\frac{N_{0}u}{r} \left(1-e^{-rt}\right)$.

Taking the derivative of Eq. S2.1 with respect to *t*, we obtain the (unconditional) probability density that a mutation that appears at time *t* saves the population:

$f\left(t\right) \approx re^{-rt } C e^{-C (1-e^{-rt})}$, (S2.2)

where $C = 2N_{0}u\left(s-r\right)/r$. This density is unconditional as the total probability that a beneficial mutation appears that will rescue the population may be much less than one. Normalizing Eq. S2.2 by this total probability (Eq. 1 of the main text), we obtain the desired probability density: conditional on rescue, the distribution of times at which the rescuing mutation appears is approximately

$f\left(t |rescue occurs\right) \approx re^{-rt }\frac{C e^{-C (1-e^{-rt})}}{1-e^{-C}}$. (S2.3)

When *C*, which roughly corresponds to the probability of rescue, is small, the second term of Eq. S2.3 is close to one and we are left with $f\left(t |rescue occurs\right) \approx re^{-rt }$, *i.e*., we recover the exponential distribution of waiting times of Orr and Unckless [1]. Computer simulations (see Supplemental Fig. S1) confirm that Eq. S2.3 provides an improved fit to actual origination times of rescuing mutations and that the previous exponential approximation is often roughly adequate, especially at smaller *C*.

**References**

1. Orr HA, Unckless RL (2008) Population extinction and the genetics of adaptation. American Naturalist 172:160-169.
